# The Age-Friendly Cities and Communities Questionnaire: A validation study of the Hebrew version in Israel

**DOI:** 10.1016/j.heliyon.2024.e39182

**Published:** 2024-10-11

**Authors:** Liat Ayalon, Jeroen Dikken, Joost van Hoof

**Affiliations:** a Louis and Gabi Weisfeld School of Social Work, Bar Ilan University, Ramat Gan, 52900, Israel; b Research Group of Urban Ageing, Faculty of Social Work & Education, The Hague University of Applied Sciences, Johanna Westerdijkplein 75, 2521, EN Den Haag, the Netherlands; c Faculty of Health, Nutrition & Sport, The Hague University of Applied Sciences, Johanna Westerdijkplein 75, 2521, EN Den Haag, the Netherlands; d Department of Systems Research, Faculty of Spatial Management and Landscape Architecture, Wrocław University of Environmental and Life Sciences, ul. Grunwaldzka 55, 50-357, Wrocław, Poland

**Keywords:** Psychometric, Measurement development, Validity, Reliability, Age-friendly cities

## Abstract

The present study examined the reliability and validity of the Hebrew version of the Age-Friendly Cities and Communities Questionnaire (AFCCQ-IL). The present study was conducted in all four Israeli cities, acknowledged as age-friendly during the time of the study in June–July 2023, namely: Tel Aviv-Jaffa, Herzliya, Kfar Saba, and Jerusalem. A total of 223 Hebrew speakers over the age of 65 participated in the study. Structure validity was confirmed via confirmatory factor analysis and reliability was established. Slight dissatisfaction was noted regarding respect and social inclusion. There was variability across the four cities with Jerusalem fairing worse than the other three cities. The findings are of relevance for urban planners and policy stakeholders.

## 1 Introduction

In response to the aging of the population and the increasing urbanization worldwide, the World Health Organization (WHO) has been promoting the age-friendly cities initiative starting in the early 2000 [[1], [2], [3]]. Among the major steps in advancing the field were a set of guidelines that provided informative tools for the establishment of age-friendly cities [1]. These guidelines propose ways to make cities and communities more age-friendly through community development, policy, and advocacy. This was followed by the establishment of the Global Network for Age-Friendly Cities and Communities (AFCC) in 2010. The network aims to connect cities, communities, and organizations which are devoted to facilitating active aging in the urban environment [4]. Currently (in October 2024), the network has over 1600 members. In recognition of the importance of the topic, the UN Decade of Healthy Ageing, which was launched in 2021, placed the concept of AFCC as one of four major pillars, essential for the healthy aging and active participation of older persons in society [5].

The AFCC initiative follows a holistic ecological approach, which views the fit between older persons and their environment as crucial for people’s well-being and health. Such an approach stresses the fact that a well-matched environment can significantly enhance older persons' quality of life. It recognizes that the physical and social aspects of a community can profoundly impact the health and well-being of older individuals. The WHO guidelines propose eight domains, which are thought to facilitate the participation and engagement of older persons in their respective communities [1]. Given the diversity of the aging population and the diversity of the urban environment, the exact characteristics and importance of each of these domains may vary, but they are all considered crucial to promoting active aging [6]. The new guidelines released in 2023 provide a step-by-step approach for developing national programs, including detailed examples from around the world [3]. To become age-friendly, cities and communities should follow several steps. A first step includes the engagement of relevant stakeholders, including older persons, in the assessment of the age-friendliness characteristics of the city. This is followed by strategic planning and prioritization to achieve age friendly outcomes. Next, is the implementation stage, which is followed by ongoing thorough monitoring and evaluation.

**The present study.** The present study examined the reliability and validity of the Hebrew version of the AgeFriendly Cities and Communities Questionnaire (AFCCQ-IL) [[7], [8]] in the Israeli context. The present study is important given the growing interest of cities and communities worldwide in joining the AFCC initiative [[9], [10]]. As one of the components of the AFCC initiative concerns the ongoing monitoring and evaluation, it is essential to establish psychometrically reliable and valid instruments which are suitable for use in various national and cultural contexts. The focus on older persons stems from the rationale of “nothing about us without us.” It is impossible to truly examine AFCC without directly querying older persons about their experiences.

The present study was conducted in Israel. Israel is a relatively young society, with about 12 % of its population being over the age of 65 [11]. It is highly urbanized with approximately 91 % of the population living in urban areas. The present study was conducted in all four Israeli cities, acknowledged as age-friendly during the time of the study in June–July 2023, namely: Tel-Aviv Jaffa, Herzliya, Kfar Saba, and Jerusalem. As this represents a relatively small number, it is expected that the establishment of the psychometric properties of the AFCCQ-IL will increase awareness to the topic and will provide policy stakeholders and urban planners with an easy-to-use tool to assess progress in the development of AFCC. It is important to note that the AFCCQ has already been used in several countries, representing varied geographical areas including the Netherlands [12], North Macedonia [13], Romania [14], Japan [15], and Turkey [16]. This makes the validation of the AFCCQ-IL in Israel particularly important as it provides a platform for comparison of the present results to results obtained in other countries.

## 2 Methods

### 2.1 Sample and procedure

The study was approved by the ethics committee of the first author’s university on May 2023 (#042303). All participants provided an informed consent and were financially reimbursed for their participation. Participants received a link via a survey agency and were instructed to complete the questionnaire online. Data collection took place in June and July 2023. Hebrew speakers over the age of 65 who resided in one of the four Israeli age-friendly cities were eligible to participate in the study. A total of 223 people participated in the study. The average age of the sample was 71.0 (SD = 4.5) and the majority was born in Israel (78.5 %). The sample had a large representation of women (65 %). The average number of years in the city was 40.7 (SD = 23.0). For additional details concerning the sample see Table 1. Below is a description of the four cities that participated in the study:

**Table 1:** Demographics of participants Israel (total = 223).

	Total	Herzliya (n = 22)	Jerusalem (n = 65)	Kfar Saba (n = 31)	Tel Aviv Jaffa (n = 105)
Gender					
Male	78 (35.0 %)	12 (54.5 %)	18 (23.7 %)	14 (45.2 %)	34 (32.4 %)
Female	145 (65.0 %)	10 (45.5 %)	47 (72.3 %)	17 (54.8 %)	71 (67.6 %)
**Age Mean** (SD)	71.0 (4.5)	72.7 (4.0)	69.6 (4.6)	70.1 (3.7)	71.7 (4.6)
**Born in the state of Israel**	175 (78.5 %)	20 (90.9 %)	51 (78.5 %)	19 (61.3 %)	85 (81.0 %)
**Duration of residence in the city** (years (SD))	40.7 (23.0)	35.1 (19.9)	50.4 (21.4)	29.3 (19.9)	39.3 (23.4)
**Religion**					
Secular Jew	138 (61.9 %)	12 (54.5 %)	24 (36.9 %)	19 (61.3 %)	83 (79.0 %)
Traditional Jew	36 (16.1 %)	8 (36.4 %)	13 (20.0 %)	4 (12.9 %)	11 (10.5 %)
Religious Jew	17 (7.6 %)	1 (4.5 %)	14 (21.5 %)	–	2 (1.9 %)
Ultra-Orthodox Jew	4 (1.8 %)	–	4 (6.2 %)	–	–
*Missing*	28 (23.6 %)	1 (4.5 %)	10 (15.4 %)	8 (25.8 %)	9 (8.6 %)
**Level of educational**					
Elementary or less	2 (0.9 %)	1 (4.5 %)	–	1 (3.2 %)	–
High school without matriculation certificate	23 (10.3 %)	3 (13.6 %)	11 (16.9 %)	2 (6.5 %)	7 (6.7 %)
High school with matriculation certificate	24 (10.8 %)	2 (9.1 %)	5 (7.7 %)	4 (12.9 %)	13 (12.4 %)
High school without academic degree	59 (26.5 %)	5 (22.7 %)	20 (30.8 %)	10 (32.3 %)	24 (22.9 %)
Academic degree	65 (29.1 %)	6 (27.3 %)	14 (21.5 %)	9 (29.0 %)	36 (34.3 %)
Master’s degree	50 (22.4 %)	5 (22.7 %)	15 (23.1 %)	5 (16.1 %)	25 (23.8 %)
**Type of dwelling**					
Owner-occupant	185 (83.0 %)	18 (81.8 %)	58 (89.2 %)	24 (77.4 %)	85 (81.0 %)
Private rent	38 (17.0 %)	4 (18.2 %)	7 (10.8 %)	7 (22.6 %)	20 (19.0 %)
**Cohabiting with a spouse or partner**	166 (74.4 %)	19 (86.4 %)	49 (75.4 %)	23 (74.2 %)	75 (71.4 %)
**Receiving care**	64 (28.7 %)	8 (36.4 %)	19 (29.2 %)	3 (9.7 %)	34 (32.4 %)
**Managing one or more chronic conditions**	73 (32.7 %)	6 (27.3 %)	23 (35.4 %)	12 (38.7 %)	32 (30.5 %)
**Using a walking aid**	14 (6.3 %)	1 (4.5 %)	7 (10.8 %)	2 (6.5 %)	4 (3.8 %)

**Tel Aviv-Jaffa.** This is the most populous city in the Gush Dan metropolitan area. Its population size is a little less than half a million. It has the largest economy per capita in the Middle East and the highest cost of living in the world. People over the age of 65 constitute 15.3 % of the population. Approximately 90 % of the population is Jewish. It is ranked 8 on the 10-point socioeconomic bracket (with 10 being the maximum), though it is highly diverse economically.

**Herzliya**. This is an affluent city in the central coast of Israel, a home to many of the start-up companies in the country. It has a population of a little over 100,000 people, with 19.4 % being over the age of 65. About 95 % of its population is defined as Jewish. It is ranked 8 on the socioeconomic bracket.

**Kfar Saba.** The city is in the Sharon region in the central district of Israel. It has a little more than 110, 000 habitants and is mostly Jewish. About 20.1 % of its population is over the age of 65. It is ranked 7 on the socioeconomic bracket.

**Jerusalem**. This is the capital of Israel and is one of the oldest cities in the world. It is considered holy to the three Abrahamic religions. It is home of more than 1 million people and is the largest city in the country of Israel, hosting all the governmental institutions. Only 9.4 % of its population is over the age of 65, given the large number of ultra-orthodox Jewish people and Arab large families. It is ranked 4 on the socioeconomic bracket but is highly diverse with some of its districts being ranked as low as 1. About 61 % of its population is Jewish, and the remaining is Arab (mainly Muslim).

**Fig. 1:**
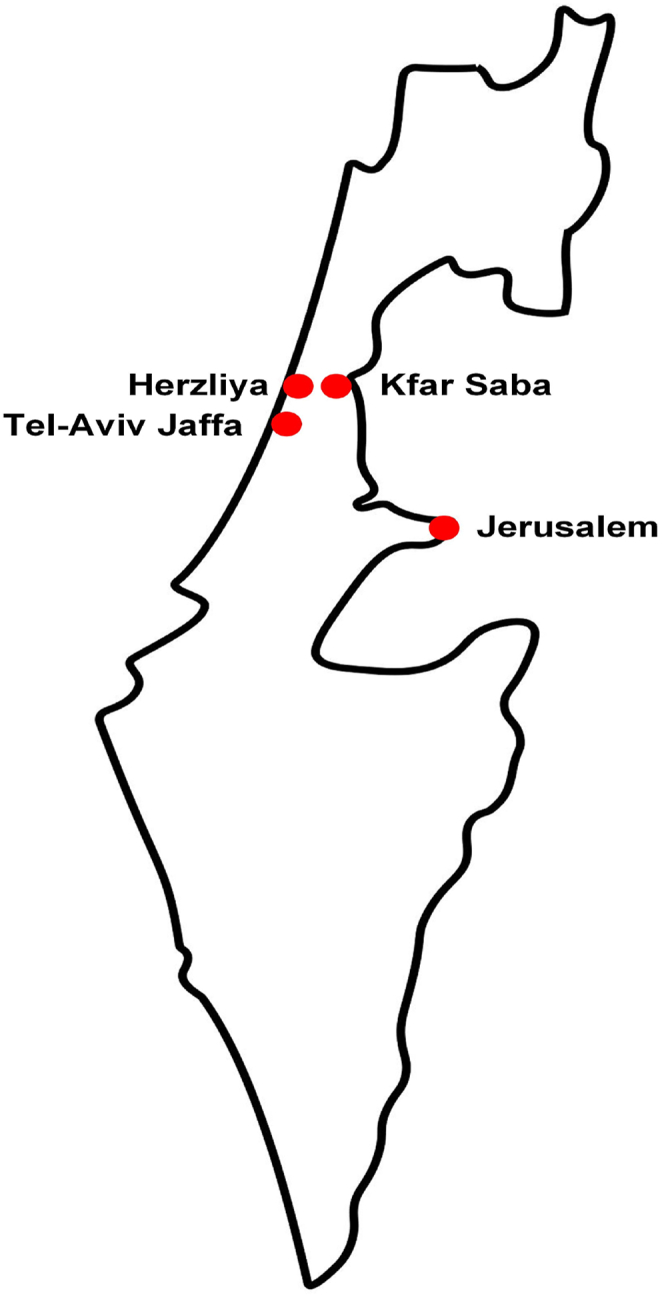
Map of Israel with the four cities. Source of the map Shutterstock stockvector-ID: 1298146159.

See a map of Israel, depicting the four cities in Fig. 1.

### 2.2 The measure

#### 2.2.1 The Age-Friendly Cities and Communities Questionnaire (AFCCQ)

The measure was developed by Dikken et al. [2020] in a rigorous and transparent manner. The psychometric properties of the measures have subsequently been evaluated and established in several other countries including Turkey, Poland, Japan, Romania, North-Macedonia, and others [[7], [17], [18]]. The final measure consists of 23 items, which cover nine domains of age-friendliness (eight by the WHO and one domain relating to finance). Items are ranked on a 5-point Likert scale, 1 = completely disagree-5 = completely agree. The higher a score on a domain the more age-friendly a city is. The measure was back translated from English to Hebrew by two independent bi-lingual researchers for use with Hebrew speakers in Israel (AFCCQ-IL). See Table 2.

**Table 2:**
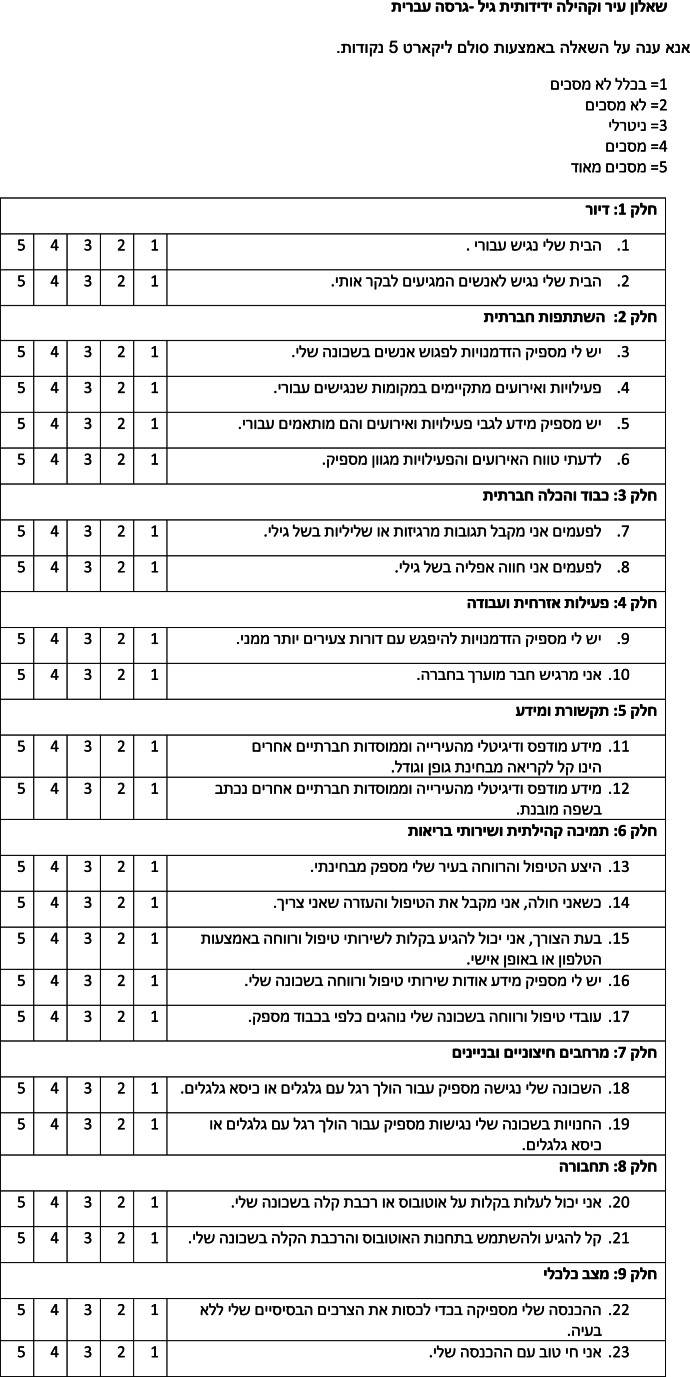
Age-Friendly Cities and Communities Questionnaire AFCCQ-IL (Hebrew version).

#### 2.2.2 Analysis

A Confirmatory Factor Analysis (CFA) was applied to test whether the factor structure of the Israeli data aligned with the model proposed by Dikken et al. [7]. By fixing the variance of the latent variables at unity, the factors were allowed to co-vary, using an approach comparable to ProMax rotation in Exploratory Factor Analysis (EFA), which assumes interrelated factors. The model’s fit was evaluated by using several indices. The normed χ^2^ was chosen for its reduced sensitivity to large sample sizes, with values of 5 or below considered acceptable [19]. To further assess model adequacy, the Comparative Fit Index (CFI) and Tucker Lewis Index (TLI) were used, where values of 0.90 or above indicate a well-fitting model [20]. Additional indices, such as the Root-Mean Squared Residual (SRMR) and Root-Mean Square Error of Approximation (RMSEA), were calculated, with acceptable cutoffs for both being less than 0.08 [21]. Together, these indices provided a well-rounded view of the model’s goodness of fit. Internal consistency of the model was established via composite reliability, a more robust metric than Cronbach’s alpha in the context of CFA. A composite reliability score of 0.70 or higher was used as the threshold to ensure that the latent constructs were measured reliably [22]. In addition, descriptive statistics, such as means and standard deviations, were calculated for each domain, examining differences across the four cities involved in the study. This helped to identify any potential regional variation in the data and contributed to a more nuanced understanding of the constructs across geographic locations. The analyses were performed using IBM SPSS Amos version 28.0 [23], which is frequently used for structural equation modeling (SEM). Amos provided a user-friendly platform for building complex models, enabling a comprehensive evaluation of both statistical fit and the practical applicability of the findings.

## 3 Results

**Fig. 2:**
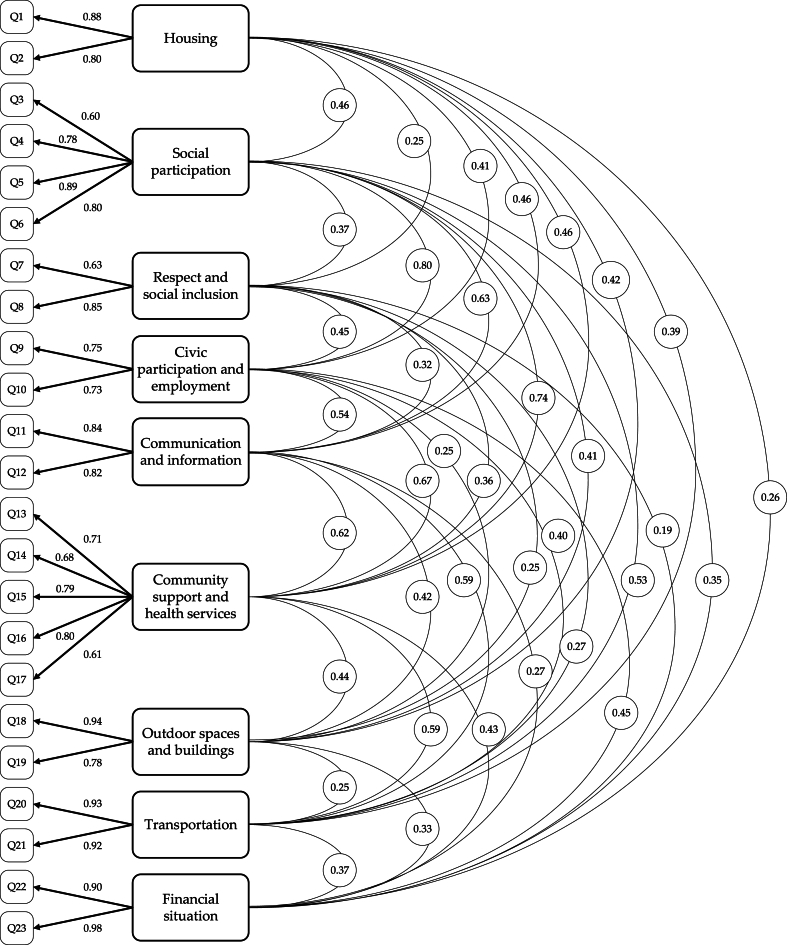
Factor structure based on the confirmatory factor analysis.

A model composed of nine dimensions was established. See Fig. 2. This model resulted in an adequate fit as can be seen in Table 3. The reliability analysis also was adequate with all dimensions having a composite reliability greater than .7. See Table 4.

**Table 3:** Fit of data from Israel with the original model as described by Dikken et al., 2020.

Model	Normed χ^2^	CFI	TLI	SRMR	RMSEA [90 % Confidence interval]
Model 1.	1.610	0.957	0.944	0.0438	0.052 [0.041–0.063]

**Table 4:** Reliability per factor of the AFCCQ-IL.

Domain	Housing	Social Participation	Respect and Social Inclusion	Civic Participation and Employment	Communication and Information	Community Support and Health Services	Outdoor Spaces and Buildings	Transportation	Financial Situation
**Composite Reliability**	0.831	0.856	0.718	0.707	0.820	0.845	0.853	0.920	0.940

Table 5 presents the mean and standard deviation per domain across the entire sample and in each of the four cities. Overall, the total score indicated moderate satisfaction with the age-friendliness features of 3 of the cities except for the Jerusalem sample which was only slightly satisfied. Although the sample reported high satisfaction concerning housing and moderate satisfaction with social participation, civic participation and employment, and transportation, there were notable variations in other domains. Specifically, respect and social inclusion was ranked as slightly satisfactory by older persons in Herzliya and Tel Aviv-Jaffa, but as slightly dissatisfying in Kfar Saba and Jerusalem. Community support and health services were ranked as slightly satisfactory in Jerusalem but as satisfactory in the other three cities. Outdoor spaces and buildings were ranked as slightly dissatisfactory in Jerusalem but as slightly satisfactory in the other three cities. Whereas the financial situation was ranked as highly satisfactory in Herzliya and as satisfactory in Tel Aviv, it was ranked as slightly satisfactory in the other two cities [24].

**Table 5:**
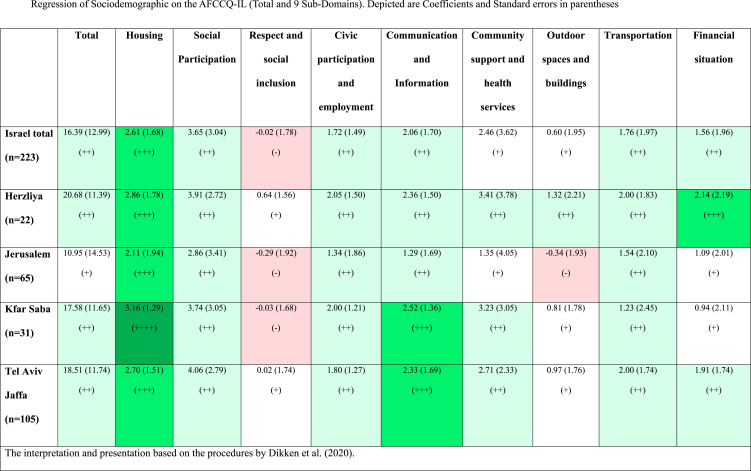
Scores (Mean + - SD) for AFCCQ-IL domains for Israel and the four participating cities (n = number of participants) following presentation guidelines as described by Dikken et al. (2020).

## 4 Discussion

The present study established the validity and reliability of the Hebrew version of the AFCCQ in Israel (i.e., the AFCCQ-IL). This is important given the need for ongoing continued monitoring of the efforts made by cities and communities to become age-friendly [25]. As currently only four cities in Israel are officially defined as age-friendly, more efforts must be put in place to develop a national age-friendly agenda and policy in line with the recent WHO [2023] [3] report on the development of national age-friendly strategies. Such efforts should rely on empirical evidence using psychometrically valid and reliable measures such as the AFCCQ.

Subsequently to establishing its validity and reliability, the AFCCQ-IL has identified several areas that should be strengthened across all four cities as well as major differences between the four Israeli age-friendly cities. One area that requires attention is the respect and social inclusion domain, ranked as slightly dissatisfactory by the entire sample and especially by older persons in Jerusalem and Kfar Saba. Compared with the other two cities that participated in the study (Tel Aviv and Herzliya), Jerusalem and Kfar Saba have lower socioeconomic status. Past research has found a relationship between the degree of social inclusion and the sociodemographic characteristics of the neighborhood. Specifically, in those neighborhoods that are going through gentrification, older persons are more likely to report social exclusion [26]. As the entire state of Israel is currently under construction boom [27], it is possible that areas of lower socioeconomic status experience even more substential changes over time.

Past research has stressed the tie between ageism and urbanization, arguing that the urban environment can foster multiple inequalities associated with age and aging [28]. Likewise, a study conducted in Israel has stressed the relationship between the characteristics of the neighborhood and ageism. That study has shown that higher levels of social integration of older neighborhood residents was partially explained by lower levels of ageism directed by younger neighborhood members [29]. The present study adds by pointing to a particular area that requires further attention of policy stakeholders and urban planners as older Israelis are generally dissatisfied with their level of inclusion in Israeli society.

Another notable domain is that of outdoor spaces and the built environment, which was ranked as slightly satisfactory overall, but as slightly dissatisfactory in Jerusalem. Hence, although older persons expressed high levels of satisfaction with their own home environment, they had reservations concerning their outdoor environment. As people age, the nearby environment and the home become increasingly important [30]. Hence, more needs to be done to improve the outdoor environments so that they provide a better fit to the needs of Israeli older persons.

Several limitations concerning the present study should be mentioned. First, we only validated the measure for use with Hebrew speakers. Future studies will benefit from establishing the psychometric properties of the AFCCQ-IL for use with Arab and Russian speakers in Israel. A Russian language version for use within the Russian Federation has been developed in 2022–2023 building on the work concerning age-friendly health care by Ziganshina et al. [31], but this version of the tool is not validated for use in the Israeli context. It was, however, validated in the multi-ethnic and multi-religious city of Kazan in the Republic of Tatarstan, with a mixed Christian and Muslim population. The AFCCQ has also been validated for use in the multi-ethnic and multi-religious city of Skopje, with a mixed ethnic population of Macedonians and Albanians. Despite small but significant differences between the older populations of both ethnicities, the instrument proved valid for use in North Macedonia [32]. In the context of Israel, a future Arabic language tool could also be used for Arab older persons in the country, which in some areas, for instance, in the Galilea, are a sizeable minority and may have different views regarding the age-friendliness of their rural residence. In addition, an Arab-language version of the AFCCQ-IL could serve as a steppingstone for validation in other Arabic-speaking nations (for instance, the United Arab Emirates and its Emirate of Sharjah having joined the Global Network), recognizing that Arabic is also one of the six working languages of the United Nations.

The fact that data collection relied on online administration also is notable. It is highly likely that those who completed the online survey are more educated and have a stronger connection with the community, compared with those who were unable to take an online survey. In fact, we were unable to validate the Arab and Russian versions in Israel using an online survey in this stage of the research, because these populations are less likely to rely on digital technology for communication.

When considering this limitation, it is important to note that there are substantial differences between the Jewish and the Arab population of Israel. Moreover, even within the Jewish community, there are substantial differences between veteran older persons versus immigrants from the former Soviet Union [11]. These differences manifest not only in relation to well-being, quality of life and health, but also in relation to living arrangements, the outdoor environment, and socioeconomic status, with the veteran Jewish population tending to score most favorably on all of these indicators compared with the other two population groups [[33], [34]]. These differences are expected to also result in differential response patterns to the AFCCQ-IL.

In addition, although we obtained data from all four cities, the samples are not equal and there is an oversample of people from Tel Aviv-Jaffa. Results are therefore not representative on city level. This, however, was not the aim of the present study. The number of participants is sufficient to psychometrically validate the use of the AFCCQ-IL with Hebrew speakers in Israel, and, although results should be assessed with prudence, several points which require further attention by city planners and policy stakeholders alike are shown. As the findings were obtained regarding the four age-friendly Israeli cities, it is important to examine other Israeli cities to see how they compare to the officially designated age-friendly cities. These findings can provide valuable information concerning the value of the AFCC initiative and can assist in further developing an AFCC initiative at the national level. It also will be useful to assess the AFCCQ-IL against other measures of AFCC used, such as the Age-Friendly Community Survey [35] or the Age-Friendly Community Evidence-Based Tool [36].

Date collection in this study occurred several months prior to October 7, 2023. Hence, it was not affected by the horrific massacre and the ongoing war that followed. The Israeli society was changed dramatically since then. At the time of revising this paper, almost 10 months after the massacre, Israel is still at war. Moreover, there are still 101 people held hostage in Gaza, most of whom are civilians, some are over the age of 65. In addition, more than 100,000 people are still internally displaced given the ongoing war that takes place in the areas surrounding the Gaza strip and the Northern parts of the state of Israel. Many of those who are internally displaced are older persons, who are experiencing the fading of their dream to settle the country. Others have lost their loved ones during the massacre or the war that followed [[37], [38]]. It is highly likely that many of the domains assessed by the AFCCQ-IL have changed for good and further research is needed to better understand the impact of these horrific events on older Israelis.

## 5 Conclusions and recommendations

The present study provides valuable insights for researchers and policy stakeholders. We established the validity and applicability of the AFCCQ-IL for use in Hebrew. The findings underscored the overall moderate satisfaction with age-friendliness features across four Israeli cities, with specific areas such as respect and social inclusion and outdoor spaces needing improvement, particularly in Jerusalem and Kfar Saba. These findings are of relevance for urban planners and policy stakeholders because they point to the fact that a useful tool exists to assess the experiences of older persons in their nearby living environment. Moreover, the tool points to several areas that require particular attention, given the fact that they do not fully meet the expectations of older Israelis. Variations across cities are particularly notable and require further inquiry, possibly via qualitative interviews to better understand the characteristics of the environment which are possible responsible for these differences in experience. The limitations of the study, including the need for validation in other languages (Arabic and Russian) in Israel and the major impact of the digital divide on our ability to reach out to these populations, suggest future research directions. The results stress the importance of a national age-friendly agenda, aligned with WHO guidelines, to enhance the quality of life for older Israelis, while recognizing the profound challenges currently faced by the Israeli population which is undergoing the most devastating war in its recent history.

## CRediT authorship contribution statement

**Liat Ayalon:** Writing – original draft. **Jeroen Dikken:** Writing – review & editing, Formal analysis, Conceptualization. **Joost van Hoof:** Writing – review & editing, Funding acquisition, Conceptualization.

## Data availability

Data are available upon request.

## Helsinki approval

The study was approved by the ethics committee of the first author’s university on May 2023 (#042303).

## Declaration of competing interest

The authors declare that they have no known competing financial interests or personal relationships that could have appeared to influence the work reported in this paper.
